# MRI-based radiomics models predict cystic brain radionecrosis of nasopharyngeal carcinoma after intensity modulated radiotherapy

**DOI:** 10.3389/fneur.2024.1344324

**Published:** 2024-05-30

**Authors:** Jing Hou, Yun He, Handong Li, Qiang Lu, Huashan Lin, Biao Zeng, Chuanmiao Xie, Xiaoping Yu

**Affiliations:** ^1^Department of Diagnostic Radiology, Hunan Cancer Hospital and The Affiliated Cancer Hospital of Xiangya School of Medicine, Central South University, Changsha, Hunan, China; ^2^Department of Radiation Oncology, State Key Laboratory of Oncology in South China, Collaborative Innovation Center for Cancer Medicine, Sun Yat-sen University Cancer Center, Guangzhou, Guangdong, China; ^3^Department of Pharmaceuticals Diagnosis, GE Healthcare, Changsha, China; ^4^Department of Radiotherapy, Hunan Cancer Hospital and The Affiliated Cancer Hospital of Xiangya School of Medicine, Central South University, Changsha, Hunan, China

**Keywords:** nasopharyngeal carcinoma, cystic brain radionecrosis, magnetic resonance imaging, radiomics, intensity modulated radiotherapy

## Abstract

**Objective:**

To construct radiomics models based on MRI at different time points for the early prediction of cystic brain radionecrosis (CBRN) for nasopharyngeal carcinoma (NPC).

**Methods:**

A total of 202 injured temporal lobes from 155 NPC patients with radiotherapy-induced temporal lobe injury (RTLI) after intensity modulated radiotherapy (IMRT) were included in the study. All the injured lobes were randomly divided into the training (*n* = 143) and validation (*n* = 59) sets. Radiomics models were constructed by using features extracted from T2WI at two different time points: at the end of IMRT (post-IMRT) and the first-detected RTLI (first-RTLI). A delta-radiomics feature was defined as the percentage change in a radiomics feature from post-IMRT to first-RTLI. The radiomics nomogram was constructed by combining clinical risk factors and radiomics signatures using multivariate logistic regression analysis. Predictive performance was evaluated using area under the curve (AUC) from receiver operating characteristic analysis and decision curve analysis (DCA).

**Results:**

The post-IMRT, first-RTLI, and delta-radiomics models yielded AUC values of 0.84 (95% CI: 0.76–0.92), 0.86 (95% CI: 0.78–0.94), and 0.77 (95% CI: 0.67–0.87), respectively. The nomogram exhibited the highest AUC of 0.91 (95% CI: 0.85–0.97) and sensitivity of 0.82 compared to any single radiomics model. From the DCA, the nomogram model provided more clinical benefit than the radiomics models or clinical model.

**Conclusion:**

The radiomics nomogram model combining clinical factors and radiomics signatures based on MRI at different time points after radiotherapy showed excellent prediction potential for CBRN in patients with NPC.

## 1 Introduction

Radiotherapy remains the mainstay of treatment for nasopharyngeal carcinoma (NPC) due to its complicated anatomic location and unique radiotherapy-sensitivity (1). Since NPC often represents close proximity and infiltration to skull base, temporal lobes are inevitably included into the target volume, which will impose high radiation dose on brain tissue. Radiotherapy-induced temporal lobe injury (RTLI) is one of the late-latency and most serious complications (2, 3). White matter lesions (WMLs), contrast-enhanced lesions (CELs) and cystic brain radionecrosis (CBRN) are considered as three types of MRI manifestations of RTLI (4). WMLs and CELs are the most common patterns of RTLI and can be reversible, whereas CBRN is the least frequent injury pattern arising in the late stage of RTLI and rarely can be reversible (4, 5). According to a previous report (6), the occurrence of CBRN was about one-tenth of all the RTLI. Although rare, CBRN is likely to be life-threatening with increasing intracranial pressure related to mass effect and developing brain necrosis (7). Furthermore, corticosteroid, as the primary treatment for RTLI, is unlikely to provide significantly clinical benefit when liquefaction necrosis develops extensively (6, 7). In addition, the role of surgery for CBRN is limited by the bilaterality of the involvement for NPC patients. Therefore, early prediction of CBRN may be particularly important for treatment decision making and adjustment.

Currently, the imaging diagnosis of RTLI mainly depends on MRI. However, existing conventional magnetic resonance imaging (MRI) techniques can only differentiate RTLI at the relatively late stage. Radiomics turns the deep-seated feature information hidden in conventional medical images into quantitative data invisible to naked eyes (8, 9). At present, there have been several studies that use MRI at different time points to construct radiomics models for predicting RTLI in NPC (10–16). Some studies have developed radiomics nomogram models based on MRI at the end of intensity modulated radiotherapy (IMRT) to predict the RTLI in NPC patients, and these models have shown outstanding predictive performance (11, 13, 15). Zhang Bin et al. established MRI-based radiomics models at three time points before RTLI confirmation to early predict RTLI (14). Their results revealed that the model constructed based on T2WI nearest to the first time point of RTLI confirmation had the highest prediction efficiency compared with the other two models which were far from the first time point of RTLI confirmation. As far as we know, there seems to be no report that surveyed the potential of MRI-based radiomics model for the early prediction of CBRN. As the most serious type of RTLI, CBRN is clinically warranted to be predicted as early as possible in order to adjust the treatment decision and make timely clinical intervention. However, it is now unknown which time point is the earliest and most appropriate for predicting CBRN.

Therefore, the purpose of this study was to investigate the efficacy of MRI-based radiomics models in predicting CBRN in patients with NPC, as well as to determine the optimal time point for predicting the occurrence of CBRN.

## 2 Materials and methods

### 2.1 Study design and patients

This study was approved by the institutional review board of the two participating hospitals (approval numbers: KYJJ-2021-095 and B2020-417-Y01). Due to its retrospective nature, written informed consent was waived. A total of 44 patients with CBRN, involving 53 temporal lobes, were included in this study from Hunan Cancer Hospital between January 2014 and December 2021 and Sun Yat-Sen University Cancer Center between January 2011 and December 2021. To reduce the imbalance between CBRN and non-CBRN samples, 111 non-CBRN samples with 149 injured lobes WMLs and/or CELs were selected randomly. Finally, 155 eligible patients with 202 injured temporal lobes were included in this study.

The inclusion criteria were as follows: (1) pathologically confirmed diagnosis of NPC, (2) received IMRT, and (3) confirmed presence of RTLI through careful review of follow-up MRI images of the head and neck. Patients were excluded if they had (1) other abnormalities in the central nervous system, such as cerebral infarctions, tumors, infections, or NPC invasion into the middle cranial fossa, (2) no regular follow up MRI data, or (3) had CBRN at the first MRI-detected RTLI.

We extracted the patient’s clinical data from the picture archiving and communication system (PACS). Patient data included age, sex, latency period, hypertension history, drinking history, smoking history, TNM stage, T stage, N stage, M stage, and pathological differentiation degree. Dosimetric parameters for each temporal lobe including mean dose (D_mean_), maximum dose (D_max_) and minimum dose (D_min_) were obtained from dose-volume histogram (DVH).

The clinical stages of all patients with NPC were determined according to the American Joint Committee on Cancer (AJCC) TNM classification system (17). According to the guidelines recommendation for NPC, radiotherapy alone was performed for I-II stage (T1N0, T2N0), concurrent chemoradiotherapy was for II stage (T1-2N1, T3N0), and concurrent chemoradiotherapy combined with induction/adjuvant chemotherapy was for III-IVA stages.

### 2.2 MRI appearances of RTLI

All patients enrolled in the present study received regular follow-up and MRI scans at 3 months intervals in the first year, 6 months intervals in the second year, and once every year intervals thereafter according to the NCCN guideline (18). The latency of RTLI was calculated from the end of IMRT to the date of RTLI firstly detected by MRI. For each patient, the two temporal lobes were analyzed separately. The diagnosis of CBRN is based on the presence of a round or oval well-defined lesion exhibiting very high signal intensity on T2WI, with a thin or imperceptible wall. WMLs refers to the lesion in white matter with homogeneously high signal intensity on T2WI and low signal intensity on T1WI. CELs is defined as lesion with high signal intensity on T2WI and enhancement on post-contrast T1WI with or without necrosis.

### 2.3 MRI protocols

In Hunan Cancer Hospital, the MRI examinations were performed using a 1.5-Tesla MRI scanner (Optima MR360, GE Healthcare, Milwaukee, WI) equipped with a head and neck combined coil. The MRI protocols consisted of the following sequences: (1) axial T1-weighted imaging (repetition time (TR)/echo time (TE) 580 ms/7.8 ms, slice thickness 5 mm, slice number 36, slice space 1 mm, number of excitations (NEX) 2); (2) axial T2-weighted imaging with fat suppression (TR/TE 6289 ms/85 ms, slice thickness 5 mm, slice number 36, slice space 1 mm, NEX 2); and (3) axial contrast-enhanced T1-weighted (CET1-w) spin-echo images (TR/TE 500 ms/8 ms, field of view (FOV) 22 × 22 cm, NEX 2, slice thickness 4 mm, interslice gap 0.8 mm).

In Sun Yat-Sen University Cancer Center, the MRI examinations were also performed on a 1.5-Tesla MRI canner (Signa, GE, CV/i). The protocols were as follows: (1) axial T1-weighted fast spin-echo images (TR/TE 420-450/min full, slice thickness 6 mm, slice number 36, slice space 1 mm, NEX 2); (2) axial T2-weighted fast spin-echo images with fat suppression (TR/TE 3200–3500 ms/85 ms, slice thickness 6 mm, slice number 36, slice space 1 mm, NEX 2); and (3) axial contrast-enhanced T1-weighted spin-echo images (TR/TE 320-350/min full, FOV 22 × 22 cm, NEX 2, slice thickness 6 mm, interslice gap 1 mm).

**Figure 1:**
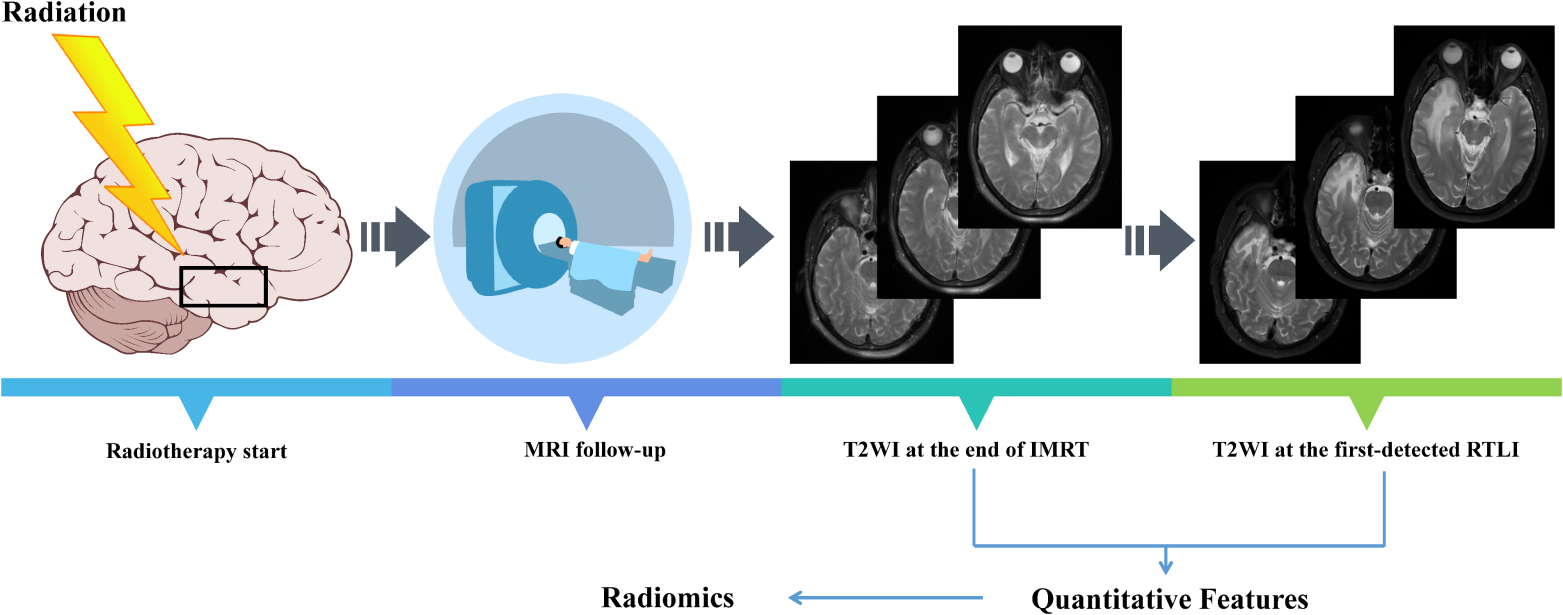
All NPC patients included in the study underwent regular MRI follow-up. NPC, nasopharyngeal carcinoma; IMRT, intensity modulated radiotherapy; RTLI, radiotherapy-induced temporal lobe injury.

For radiomics analysis, the axial T2WI images at the end of IMRT (post-IMRT) and the first-detected RTLI (first-RTLI) were used to construct CBRN prediction model (Figure 1). In addition, delta-radiomics feature was defined as the percentage change in a radiomics feature from post-IMRT to first-RTLI.

### 2.4 MRI pre-processing, segmentation, and feature extraction

To minimize heterogeneity and differences in MRI images across different institutions, pre-processing was conducted. MRI pre-processing was performed using AK software (Analysis Kit, GE Healthcare), which has been registered and approved. The preprocessing steps included resampling, skull stripping, and intensity standardization. The image resolution for this study was adjusted to 1 mm × 1 mm × 1 mm through resampling. The slice thickness of all MRI images was converted to 1 mm, through the linear difference value. Non-brain tissues were removed from the T2WI images through skull stripping. Then, image gray unified adjustment to 0–255 was done for standardization.

T2WI segmentation was performed by two radiologists (radiologists A and B, with 7 years and 12 years of experience in head and neck MRI, respectively) using ITK-SNAP software (version 3.6.0)^1^ to sketch region of interests (ROIs). Both radiologists were blinded to the CBRN results. They independently delineated along the boundaries of the white matter of bilateral temporal lobes layer by layer from the lowest to midbrain levels. Subsequently, these ROIs were automatically converted to volume of interests (VOIs) and saved as NII format file.

Inter-and intra-class correlation coefficients (ICCs) was applied to assess the reproducibility of intra-observer and inter-observer segmentation. Two abovementioned radiologists randomly drew 50 temporal lobes independently. After 1 month, radiologist A repeated the same procedure again. An ICC greater than 0.75 was considered as good consistency. A good ICC result was obtained in our study and all the rest of the images were segmented by radiologist A.

396 radiomics features were extracted from each VOI via AK software. These features were involved with six categories, including six types of texture parameters, i.e., histogram, gray-level size zone matrix (GLSZM), formfactor, haralick, gray-level co-occurrence matrix (GLCM) and run-length matrix (RLM).

### 2.5 Feature selection and radiomics signature construction

All temporal lobes were randomly divided into the training and validation sets in a proportion of 7:3. Two feature selection methods, namely the minimum redundancy maximum relevance (mRMR) (19) and the least absolute shrinkage and selection operator (LASSO), were applied to select the most predictive features in the training set (20). Firstly, mRMR method was applied to eliminate the redundant and irrelevant features according to their relevance-redundancy indexes rank upon a heuristic scoring criterion. In the mRMR algorithm, the heuristic scoring criterion typically consists of two parts, namely Maximum Relevance (MR) and Minimum Redundancy (MR). The former evaluates the relevance of each feature to the target variable, and the latter evaluates the redundancy among the selected features. If two features provide similar information, they are considered redundant. After mRMR, the top 20 features with high relevance were retained. Next, LASSO classifiers were performed to choose the optimized subset of features, and 10-fold cross-validation was applied to avoid overfitting. Through LASSO regression, the coefficients for each feature can be obtained. The radiomics signature (radscore) was calculated by summing the selected the texture features that were weighted by their respective coefficients.

### 2.6 Radiomics nomogram construction and validation

For clinical variables, firstly, univariate logistic analysis was carried out to determine the characteristics with significant association with CBRN. Variables with *P* greater than 0.05 in univariable analysis were excluded. Then, variables significantly associated with CBRN in univariate analysis were subsequently subjected to the stepwise multivariate logistic regression analysis applying the minimum value of Akaike’s information criterion (AIC) as the stopping rule. Finally, to construct the radiomics nomogram, the significant clinical variables and the radscores were introduced into the multivariate logistic regression analysis.

**Figure 2:**
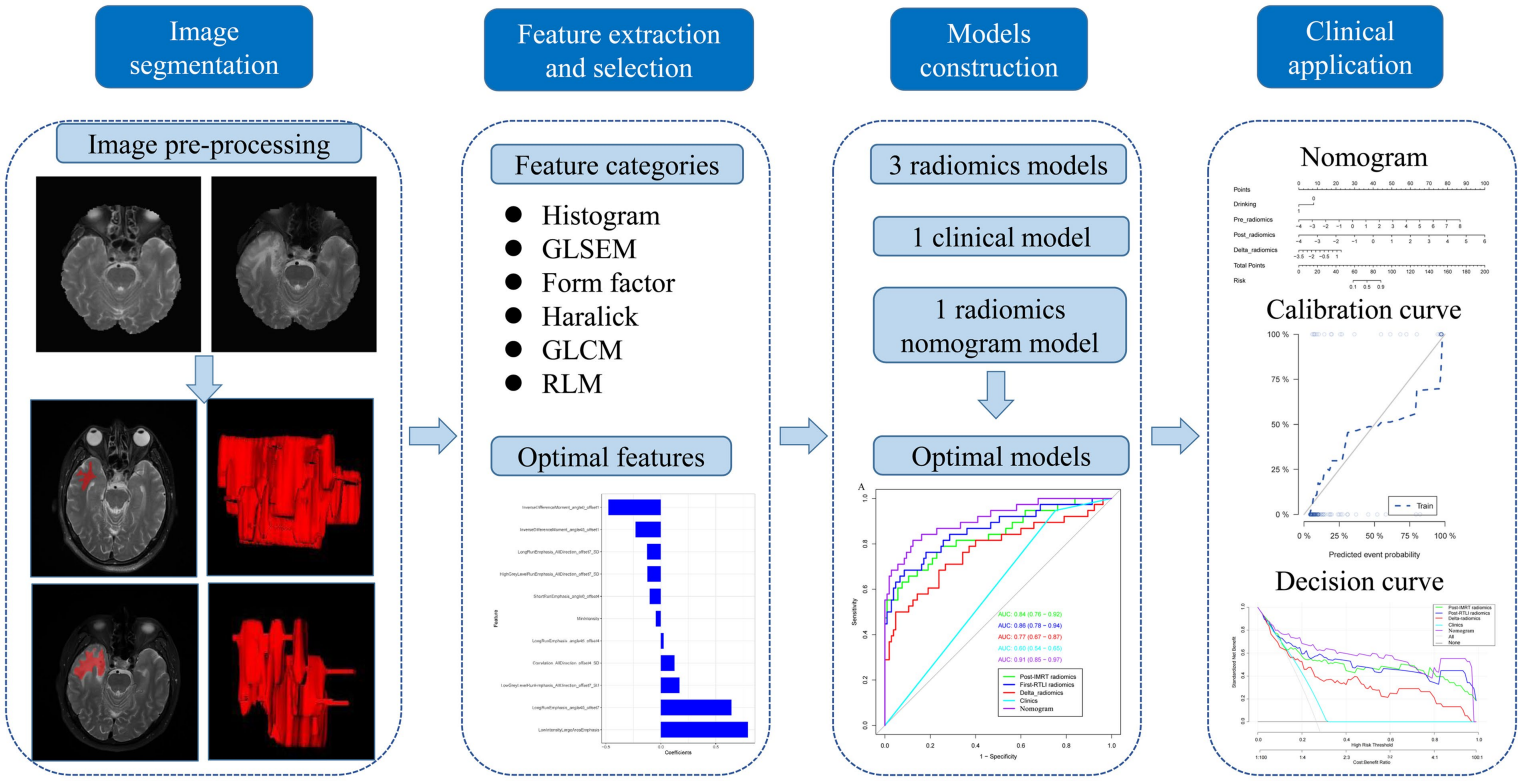
Flow chart of the radiomics analysis.

The area under the curve (AUC) value based on receiver operating characteristic (ROC) curve analysis was used to quantificationally evaluate the predictive performance of the clinical, radiomics and radiomics nomogram models. A calibration curve was generated to estimate the performance of the radiomics nomogram model. Hosmer-Lemeshow test was applied to investigative the goodness-of-fit of the radiomics nomogram model. The clinical significance of individual prediction model was evaluated by decision curve analysis (DCA), which quantifies the net benefits at different threshold probabilities in the training and validation sets. The workflow of the radiomics analysis is shown in Figure 2.

### 2.7 Statistical analysis

All the statistical analyses of the study were conducted by the R software (version 3.3.3).^2^ In two-tailed analysis, a *p* value less than 0.05 was defined as statistically significant. Mann–Whitney U test and Chi-square test were used to evaluate the continuous variables and categorical variables between groups, respectively. The univariate and multivariate logistic regression analysis were performed to identity the independent clinical predictors. The predictive performances of all models were estimated by AUC value, sensitivity and specificity based on ROC curve. The AUC values among different models were compared by DeLong’s test. The sensitivity and specificity values among different radiomics models were compared by McNemar test.

## 3 Results

### 3.1 Baseline characteristics of the patients

155 RTLI patients were recruited in the present study (121 men and 34women; mean age 43 ± 12.7 years). The median latency time from IMRT completion to the first MRI detection of RTLI and CBRN were 31 months (range, 16–63 months), 49 months (range, 33–109 months), respectively. Among the 155 RTLI patients, 44 cases were diagnosed as CBRN (bilateral, 9; unilateral, 35), and 111 cases were diagnosed as non-CBRN (bilateral, 38; unilateral, 73). The numbers of CBRN and non-CBRN lobes were 53 and 149, respectively. The patient characteristics are summarized in Table 1. These lobes were randomly divided into training (*n* = 143) and validation (*n* = 59) sets. In the training set, there were 38 CBRN lobes and 105 non-CBRN lobes. In the validation set, there were 15 CBRN lobes and 44 non-CBRN lobes. There were not significant differences in sex, year, smoking history, drinking history, hypertension history, pathological differentiation, clinical stages, D_min_, D_max_, and D_mean_ between the training and validation groups (Table 1).

**Table 1:** Basic characteristics of RTLI patients and temporal lobes in the training and validation sets.

Characteristics	No. of patients (*N* = 155)	No. of temporal lobes		*p*
		Training (*N* = 143)	Validation (*N* = 59)	
Sex				
Male	121 (78.06)	117 (81.58)	44 (73.3)	0.331
Female	34 (21.94)	26 (18.2)	15 (25.4)	
Age (mean ± sd) years	43.0 (12.7)	49.4 (9.0)	49.5 (9.3)	0.965
Smoking (mean ± sd) years	10.3 (13.0)	11 (13.2)	8.6 (12.3)	0.238
Drinking (mean ± sd) years	3.9 (9.7)	4.5 (10.7)	2.6 (6.6)	0.205
Hypertension (mean ± sd) years	0.5 (1.9)	0.5 (1.8)	0.3 (1.5)	0.262
Differentiation degree				
Undifferentiated	90 (58.0)	82 (57.3)	36 (61.0)	
Differentiated	65 (42.0)	61 (42.7)	23 (39.0)	0.630
Pathological type				
II/III	153 (98.7)	142 (99.3)	58 (98.3)	
I	2 (1.3)	1 (0.7)	1 (1.7)	0.495
TNM stage				
I	1 (0.6)	1 (0.7)	0 (0.0)	
II	4 (2.6)	1 (0.7)	3 (5.1)	
III	51 (32.9)	48 (33.6)	22 (37.3)	
IV	99 (63.9)	93 (65.0)	34 (57.6)	0.147
T stage				
T1	7 (4.5)	6 (4.2)	4 (6.8)	
T2	24 (15.5)	24 (16.8)	10 (16.9)	
T3	38 (24.5)	36 (25.2)	16 (27.1)	
T4	86 (55.5)	77 (53.8)	29 (49.2)	0.849
N stage				
N0	10 (6.5)	9 (6.3)	5 (8.5)	
N1	31 (20.0)	24 (16.8)	9 (15.3)	
N2	92 (59.3)	87 (60.8)	36 (61.0)	
N3	22 (14.2)	23 (16.1)	9 (15.3)	0.947
M stage				
M0	152 (98.1)	139 (97.20)	57 (96.6)	
M1	3 (1.9)	4 (2.80)	2 (3.4)	1.000
D_min_ (Gy)	2.6 (1.7)	2.7 (1.8)	2.5 (1.3)	0.411
D_max_ (Gy)	73.8 (9.7)	73.6 (9)	74.3 (11.3)	0.629
D_mean_ (Gy)	22.1 (7.1)	21.8 (7.2)	22.8 (7.1)	0.370

NPC, nasopharyngeal carcinoma; RTLI, radiotherapy-induced temporal lobe injury; D_max_, maximum dose; D_min_, minimum dose; D_mean_, mean dose. *P*-values are calculated using T-tests or U-tests for quantitative data, and chi-square tests for qualitative data.

The intra-reader ICC between the two measurements by radiologist A ranged from 0.776 to 0.918. The inter-reader ICC between the two radiologists ranged from 0.814 to 0.905. These results indicated a favorable inter-and intra-observer reproducibility for feature extraction.

### 3.2 Clinical model

Univariate and multivariate logistic regression analysis were used to identity the independent predictors among all the clinical variables. The univariate logistic analysis revealed that N stage (*p* = 0.002), D_mean_ (*p* = 0.045), and drinking (*p* = 0.003) were significantly associated with CBRN and were retained for further analysis. Following stepwise multivariate logistic regression analysis with AIC, only drinking history (OR: 0.17, 95% CI: 0.04–0.75, *p* = 0.019) was retained as the independent predictor of CBRN (Supplementary Table 1). The clinical model had poor predictive performance, with an AUC value of 0.60 (95%CI: 0.54–0.65) and 0.60 (95%CI: 0.54–0.66) in the training cohorts and validation cohorts, respectively.

### 3.3 Post-IMRT, first-RTLI, and delta-radiomics model

396 texture features were extracted by AK software for each temporal lobe. After mRMR and LASSO procedure for feature selection, 11, 10 and 7 features were eventually retained to construct the final post-IMRT, first-RTLI, and delta-radiomics models, respectively (Supplementary Figures 1–3). The calculation formulas of radscore were presented in the Supplementary material.

The median of radscore for the CBRN group was significantly higher than that for the non-CBRN group in the training for the post-IMRT, first-RTLI, and delta-radiomics models, respectively (0.332 vs. −1.671, *p* < 0.001; −0.062 vs. −1.677, *p* < 0.001; −0.617 vs. −1.289, *p* < 0.001). The significant differences were also found in the validation cohorts for the post-IMRT, first-RTLI, and delta-radiomics models (0.989 vs. −1.644, *p* < 0.001; −0.862 vs. −1.595, *p* < 0.001; −0.498 vs. −1.208, *p* < 0.001) respectively (Supplementary Tables 2–4 and Supplementary Figures 4–6).

In the training cohorts, the optimal post-IMRT, first-RTLI, and delta-radiomics models yielded AUC values of 0.84 (95% CI: 0.76–0.92), 0.86 (95% CI: 0.78–0.94), and 0.77 (95% CI: 0.67–0.87), respectively. In the validation cohorts, the corresponding AUC value was 0.86 (95% CI: 0.74–0.98), 0.83 (95% CI: 0.67–1.00), and 0.73 (95% CI: 0.55–0.91). The threshold values of the post-IMRT, first-RTLI, delta-radiomics models are −0.936, −1.051 and −0.573, respectively. There were no significant differences in the AUCs of the three radiomics models, in either the training or validation cohorts (*p* > 0.05).

### 3.4 Nomogram model

**Figure 3:**
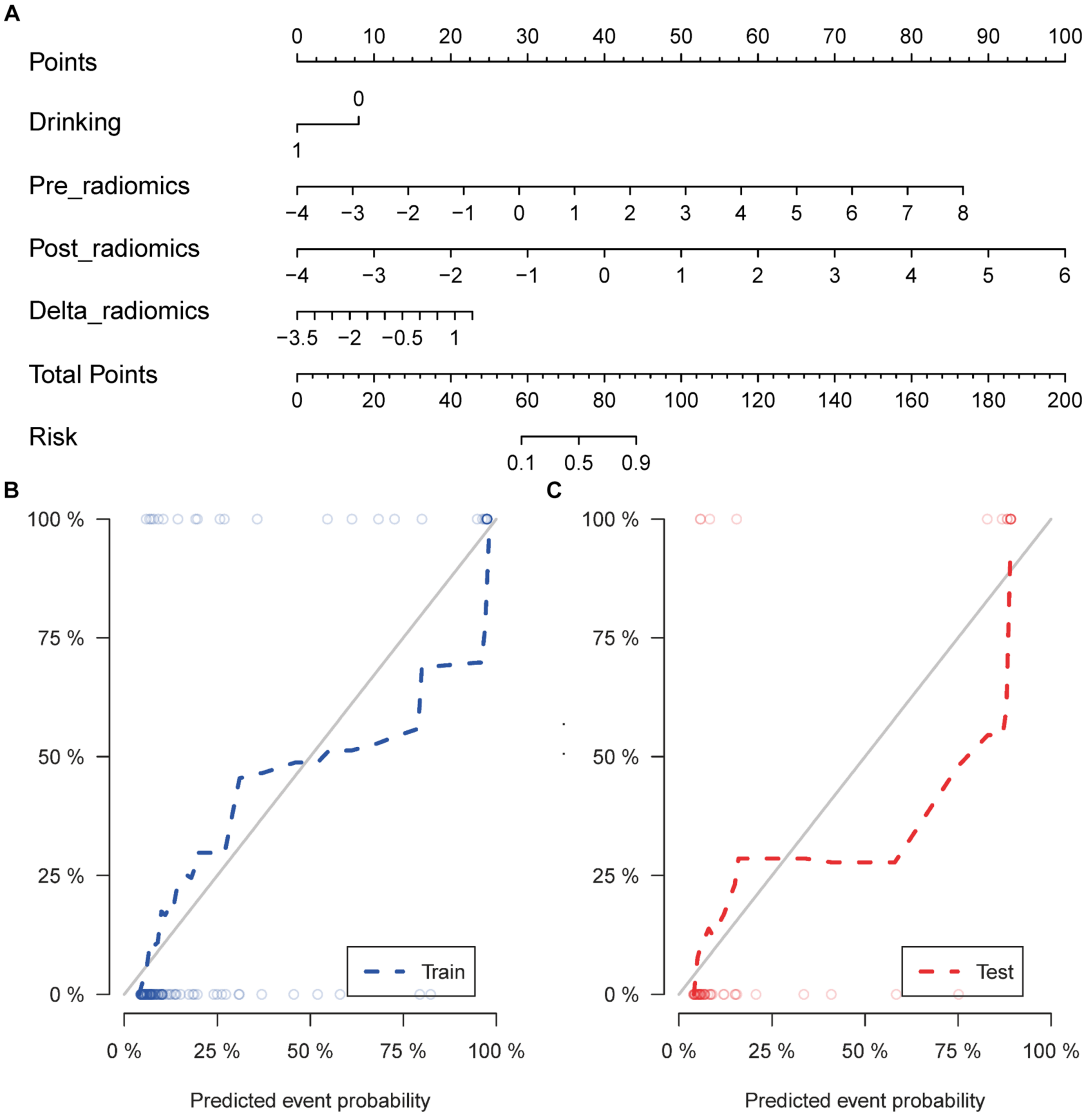
Nomogram for the prediction of CBRN in patients with NPC **(A)**. Calibration curve of nomogram model in the training **(B)** and validation sets **(C)**. CBRN, cystic brain radionecrosis; NPC, nasopharyngeal carcinoma.

**Figure 4:**
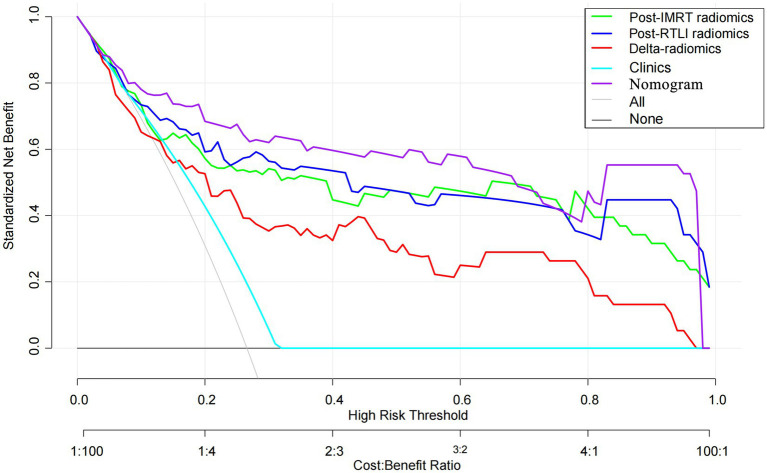
Decision curve analysis for the radiomics, clinical and radiomics nomogram models for the prediction of CBRN in patients with NPC. CBRN, cystic brain radionecrosis; NPC, nasopharyngeal carcinoma.

The calculation formula for the nomogram is also presented in the Supplementary material. The nomogram model that incorporated the above independent clinical predictors and radiomics signatures is presented in Figure 3A. The calibration curve of the nomogram demonstrated good calibration performance in both the training and validation sets at the end of IMRT and the first-detected RTLI (Figures 3B,C). The Hosmer-Lemeshow test yielded no significant difference for the nomogram model in both the training and validation sets (*p* > 0.05), indicating favorable agreement between the predicted and actual results. The decision curve analysis showed that the nomogram model provided the best performance among the five models (Figure 4).

**Figure 5:**
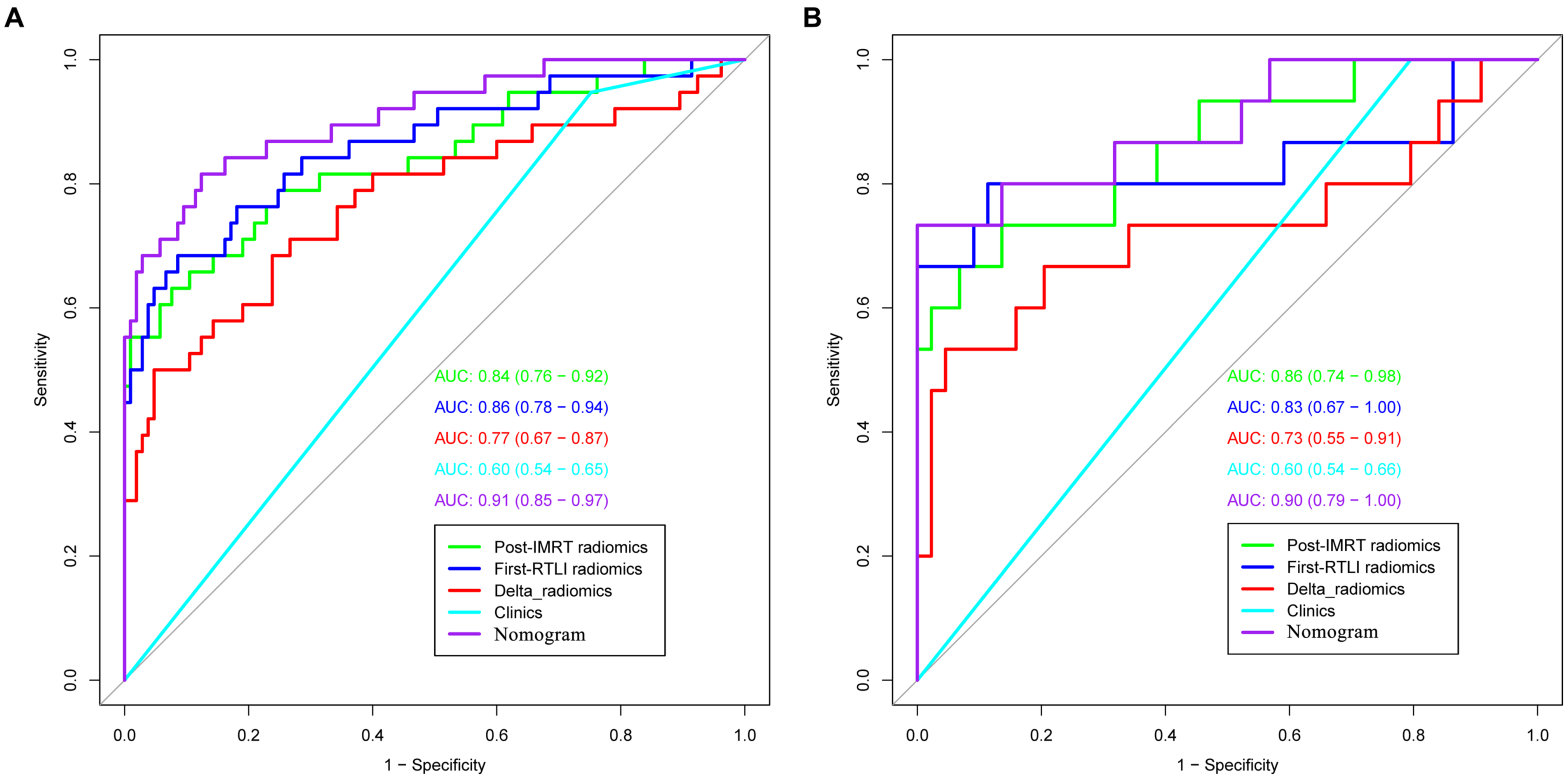
ROC curves of radiomics, clinical and radiomics nomogram models in the training **(A)** and validation sets **(B)** (the numbers in the brackets represent 95% CI). ROC, receiver operating characteristic.

ROC curves were used to evaluate the prediction efficacy of the radiomics, clinical and radiomics nomogram models in the training and validation sets (Table 2 and Figure 5). The nomogram model demonstrated superior predictive performance in the training set (AUC: 0.91, 95% CI: 0.85–0.97) as well as in the validation set (AUC: 0.90, 95% CI: 0.79–1.00). The threshold value of the nomogram model is 0.242. There were significant differences in the AUC values between the nomogram and clinical models in both the training and validation cohorts (both *p* < 0.001). Additionally, a significant difference was also found in the AUC values between the nomogram and delta radiomics models in the training cohort (*p* = 0.005), but not in the validation cohort (*p* = 0.066). However, there were no statistically significant differences in the AUC values observed between the nomogram and post-IMRT radiomics model, nor between the nomogram and first-RTLI radiomics model (*p* > 0.05). The sensitivity of the nomogram is significantly higher than that of post-IMRT (*p* = 0.002), first-RTLI (*p* = 0.002) and delta radiomics (*p* = 0.001) models. Although the specificity of the nomogram (0.88) is significantly lower than that of the post-IMRT (0.94), first-RTLI (0.97), and delta radiomics (0.95) models, it is still a good result. The accuracy of the nomogram (86.0%) is similar excellent to that of the post-IMRT (83.9%), first-RTLI (86.0%), and delta radiomics (83.2%) models.

**Table 2:** Performance of radiomics scores and radiomics nomogram in the prediction of CBRN in the training and validation sets.

Models	AUC (95%CI)	*P* value	Sensitivity	*P* value	Specificity	*P* value	Accuracy (%)
Training set							
Post-IMRT radiomics	0.84 (0.76–0.92)	0.056	0.55	0.002	0.94	0.035	83.9
First-RTLI radiomics	0.86 (0.78–0.94)	0.108	0.55	0.002	0.97	0.035	86.0
Delta radiomics	0.77 (0.67–0.87)	0.005	0.50	0.001	0.95	0.021	83.2
Radiomic nomogram	0.91 (0.85–0.97)	Ref.	0.82	Ref.	0.88	Ref.	86.0
Validation set							
Post-IMRT radiomics	0.86 (0.74–0.98)	0.541	0.60	0.083	0.93	0.025	84.7
First-RTLI radiomics	0.83 (0.67–1.00)	0.137	0.67	0.157	1	0.005	91.5
Delta radiomics	0.73 (0.55–0.91)	0.066	0.53	0.046	0.95	0.014	84.7
Radiomic nomogram	0.90 (0.79–1.00)	Ref.	0.80	Ref.	0.82	Ref.	81.3

CBRN, cystic brain radionecrosis; IMRT, intensity modulated radiotherapy; RTLI, radiotherapy-induced temporal lobe injury; AUC, area under the curve.

## 4 Discussion

In this study, we developed and validated post-IMRT, first-RTLI, delta radiomics models, as well as a nomogram model that combined clinical factors with the MRI-based radiomics signatures based on MRI at different time points after radiotherapy. The nomogram model exhibited the highest potential for predicting CBRN, although its predictive efficacy did not demonstrate significant statistical differences compared to the post-IMRT and first-RTLI radiomics models. From the DCA, the nomogram model provided more clinical benefit than the radiomics models or clinical model. In addition, the nomogram model has a significantly higher sensitivity compared to any radiomics models. Therefore, the radiomics nomogram model could predict CBRN excellently, which provide early opportunity for clinicians to make timely personalized intervention based on the predicted risk, thereby improving patient outcomes.

In this study, we observed that the post-IMRT and first-RTLI radiomics models exhibited significantly better predictive performance for CBRN compared to the clinical model. Although certain clinical variable was selected as independent prediction factors for CBRN, incorporating the clinical variable with the radiomics signatures did not significantly improve the predictive efficacy of the radiomics models, indicating that clinical factors may have limited efficacy in predicting CBRN. Notably, the final nomogram model did not include dosimetric parameters. Previous research has indicated the considerable impact of radiation dose on the occurrence of RTLI (21, 22). However, there have been noticeable variations and discrepancies in the results across different studies. For instance, some studies reported that D_max_ and D_1CC_ were independent predictors of temporal lobe necrosis (23–26). On the other hand, Wang et al. reported that only D0.5cc and D10 were reliable factors for predicting temporal lobe necrosis (27). Our previous stud demonstrated that D_max_ and D_mean_ were independent predictive indicators of RTLI (13). Additionally, Wang et al. reported that clinical parameters such as age, gender, stage, and history of diabetes and hypertension did not directly impact temporal lobe necrosis (27). Therefore, the predictive value of radiation dosimetric parameters and clinical factors in CBRN remains to be further explored.

We selected T2WI at the end of IMRT and the first-detected RTLI to construct radiomics models and radiomics nomogram for predicting the occurrence of CBRN. Our results demonstrated that both radiomics models based on MRI at different time points had similarly outstanding prediction performance for CBRN. However, our results differed somewhat from the study conducted by Zhang et al. (14), in which the model based on T2WI nearest to the time point of RTLI confirmation had the highest prediction efficiency compared with the other two models whose time points were far from RTLI confirmation. In our study, we selected the time point at the end of IMRT completion as a candidate for early prediction of CBRN based on the following consideration: when radiotherapy has finished at the end of IMRT, the externally imposed injury factor leading to RTLI has already reached its peak and will no longer increase. We hypothesized that radiotherapy-induced brain damage at the end of IMRT is different between the bilateral temporal lobes of the same patient, which may have significant impacts on the occurrence of CBRN. Encouragingly, our hypothesis was supported by the results that there were significant differences in radscore values between the CBRN and non-CBRN groups in both the training and validation sets at the end of IMRT. This suggests that clinicians may have the opportunity to predict CBRN occurrence in advance, rather than waiting for RTLI to manifest.

Radiomics approach enables the identification of imaging phenotypes and can reflect pathophysiological changes (9). We found that for a certain NPC patient, CBRN may present unilaterally instead of bilaterally during the follow-up, even though the same MRI manifestations such as WML or contrast-enhanced lesion appear bilaterally at the first detection of RTLI. Previous MRI investigations found that the evolution of RTLI may be different between the bilateral temporal lobes in the same patient (4, 5). In our speculative opinion, this might be due to the possibility that the underlying microscopic characterization of tissue has undergone different changes caused by radiotherapy even though the morphological manifestation of the initial brain injury were the same. Our speculation was supported by the results that there were significant differences in radscore values between the CBRN and non-CBRN groups in both the training and validation sets at the first-detected RTLI.

In this study, only T2WI was selected for feature extraction. The reasons are as follows: firstly, in a study by Zhang et al. (14), three radiomics models based on MRI at different times before the onset of RTLI were constructed. The study concluded that the three radiomic models using T2WI images demonstrated better predictive performance than those using CET1-w images. Secondly, we previously developed an early prediction model for RTLI in patients with NPC based on T2WI at the end of radiotherapy, which showed satisfactory predictive capabilities for RTLI (13). Additionally, we have reviewed a substantial number of studies (10–12, 15), and it is evident that some of these studies used T2WI and T1WI or CET1-w as feature extraction sequences. However, after feature selection, the final model incorporated more features from T2WI than from CET1-w. Therefore, based on these reasons, only T2WI images were selected for the construction of the radiomics model in this study. However, it is a fact that different MRI measures contain different and complementary information, CET1-w images reflect heterogeneity and architecture which are related to radiation necrosis in a histology of RTLI analysis. A combination of these measures may improve the predictive performance of CBRN. In future studies, the prediction model will be constructed by combining multiple MRI measures and exploring the best measure for predicting CBRN.

Our study has several limitations. Firstly, the number of CBRN cases was relatively small due to its low incidence rate, as well as some NPC patients who have no regular follow-up after IMRT. Secondly, the combination of MRI images from two different institutions to construct the prediction model may have been affected to a certain extent by differences in technical and protocols factors, despite the fact that all images had undergone preprocessing and standardization. Therefore, future studies are needed that include both internal and external test with larger sample sizes in multicenter observational studies.

In conclusion, we constructed and validated radiomics models and radiomics nomogram model based on T2WI at the end of IMRT and the first-detected RTLI for the early prediction of CBRN in patients with NPC. The radiomics nomogram model combining clinical factors and radiomics signatures based on MRI at different time points after radiotherapy showed excellent prediction potential for CBRN in patients with NPC.

## Data availability statement

The raw data supporting the conclusions of this article will be made available by the authors, without undue reservation.

## Ethics statement

The studies involving humans were approved by Hunan Cancer Hospital and Sun Yat-sen University Cancer Center. The studies were conducted in accordance with the local legislation and institutional requirements. Since this was a retrospective study, the ethics committee/institutional review board waived the requirement of written informed consent for participation from the participants or the participants’ legal guardians/next of kin.

## Author contributions

JH: Conceptualization, Data curation, Funding acquisition, Methodology, Software, Validation, Visualization, Writing – original draft. YH: Conceptualization, Data curation, Resources, Writing – review & editing. HaL: Conceptualization, Data curation, Formal analysis, Software, Validation, Writing – review & editing. QL: Data curation, Validation, Writing – review & editing. HuL: Writing – review & editing. BZ: Data curation, Resources, Writing – review & editing. CX: Conceptualization, Resources, Supervision, Validation, Visualization, Writing – review & editing. XY: Conceptualization, Supervision, Validation, Visualization, Writing – review & editing.

## Footnotes

^1^ www.itksnap.org

^2^ http://www.Rproject.org
