# Supplementary material for: MRI-based radiomics models predict cystic brain radionecrosis of nasopharyngeal carcinoma after intensity modulated radiotherapy
Source: Front Neurol. 2024 May 30;15:1344324. doi: 10.3389/fneur.2024.1344324 (PMC11169923; doi:10.3389/fneur.2024.1344324)
Supplement: Supplementary file 1 [file Data_Sheet_1.pdf]

Supplementary Material

Supplementary Data

Radscore and Nomoscore

The calculation formula of post-IMRT Radscore is presented as follows:

Eq. (A.1): Radscore=0.792*LowIntensityLargeAreaEmphasis+
0.640*LongRunEmphasis_angle45_offset7+
0.169*LowGreyLevelRunEmphasis_AllDirection_offset7_SD+
0.124*Correlation_AllDirection_offset4_SD+
0.025*LongRunEmphasis_angle45_offset4+
-0.047*MinIntensity+
-0.102*ShortRunEmphasis_angle0_offset4+
-0.123*HighGreyLevelRunEmphasis_AllDirection_offset7_SD+
-0.124*LongRunEmphasis_AllDirection_offset7_SD+
-0.232*InverseDifferenceMoment_angle45_offset1+
-0.476*InverseDifferenceMoment_angle0_offset1+-1.13829076187366*

The calculation formula of first-RTLI Radscore is presented as follows:

Eq. (A.2): Radscore=0.693*Inertia_angle0_offset1+
0.682*IntensityVariability+
0.327*LongRunEmphasis_angle135_offset4+
0.227*ShortRunEmphasis_AllDirection_offset7_SD+
0.194*LargeAreaEmphasis+
0.098*ZonePercentage+
0.021*LongRunEmphasis_AllDirection_offset4_SD+
0.009*LongRunEmphasis_angle45_offset4+
-0.040*LongRunEmphasis_angle90_offset7+
-0.433*ShortRunEmphasis_angle0_offset7+-1.13021739511794*

The calculation formula of delta Radscore is presented as follows:

Eq. (A.3): Radscore=0.373*InverseDifferenceMoment_angle45_offset1+
0.342*Variance+
0.145*HighGreyLevelRunEmphasis_AllDirection_offset7_SD+
0.044*LowIntensityLargeAreaEmphasis+
0.039*ShortRunEmphasis_AllDirection_offset4_SD+
0.028*Correlation_AllDirection_offset1_SD+
-0.258*SmallAreaEmphasis+-1.10430096440043*

The calculation formula of radiomics nomoscore is presented as follows:

Eq. (A.4): Nomoscore = 2.875*(Intercept)+-1.178*Drinking+
1.063*post-IMRT radiomics+
1.472* first-RTLI radiomics+
0.671*Delta radiomics

Supplementary Figures and Tables

Supplementary Figures

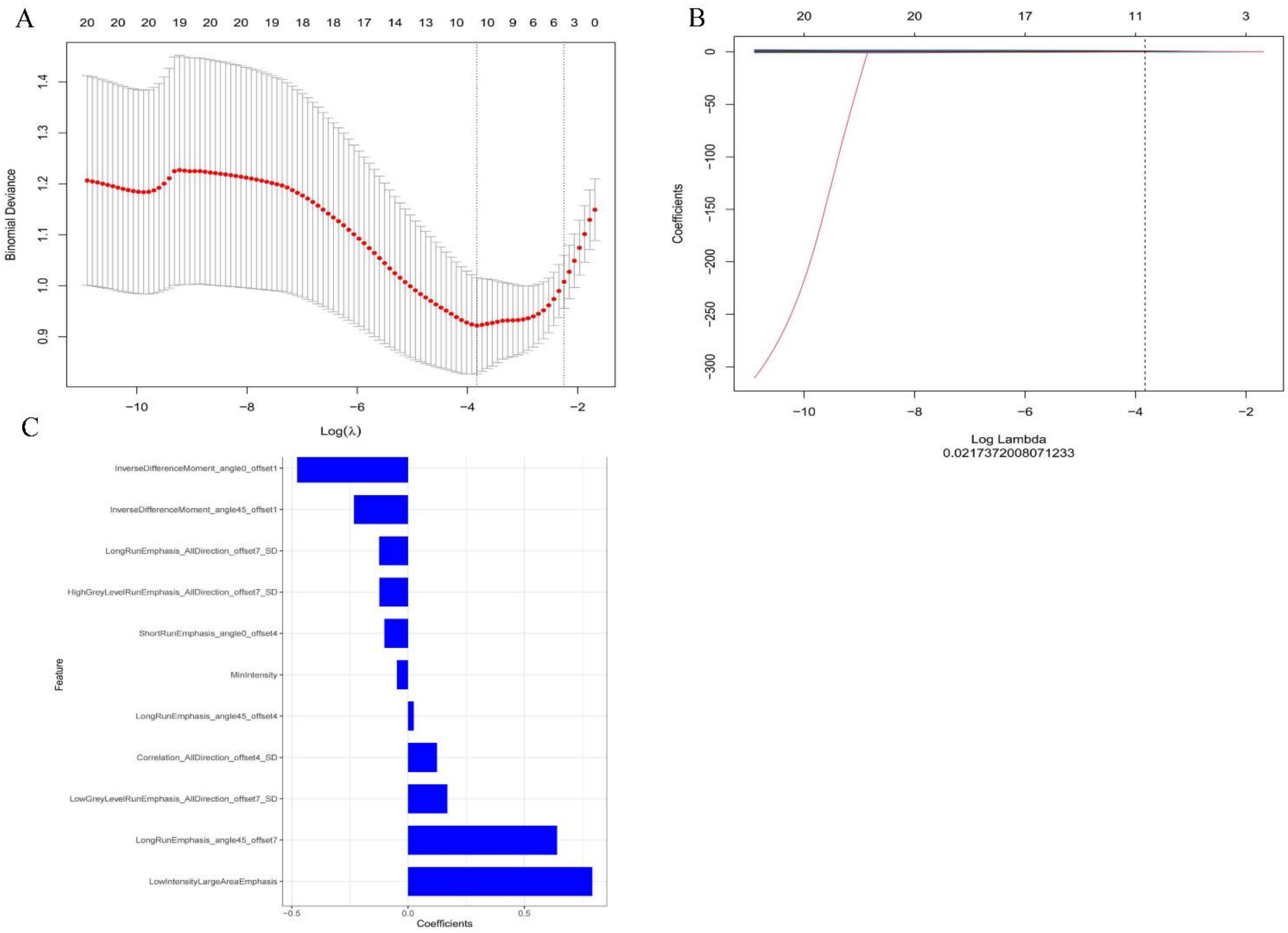

Supplementary Figure 1. Feature selection for post-IMRT radiomics model. (A) The error rate curve. (B) LASSO coefficient λ graph. Coefficient λ was selected in the LASSO using a 10-fold cross-validation. The coefficient λ with the lowest error rate was chosen. (C) The remaining features of T2WI after feature selection.

Note. IMRT = intensity modulated radiotherapy, LASSO = least absolute shrinkage and selection operator, T2WI = T2 weighted imaging.

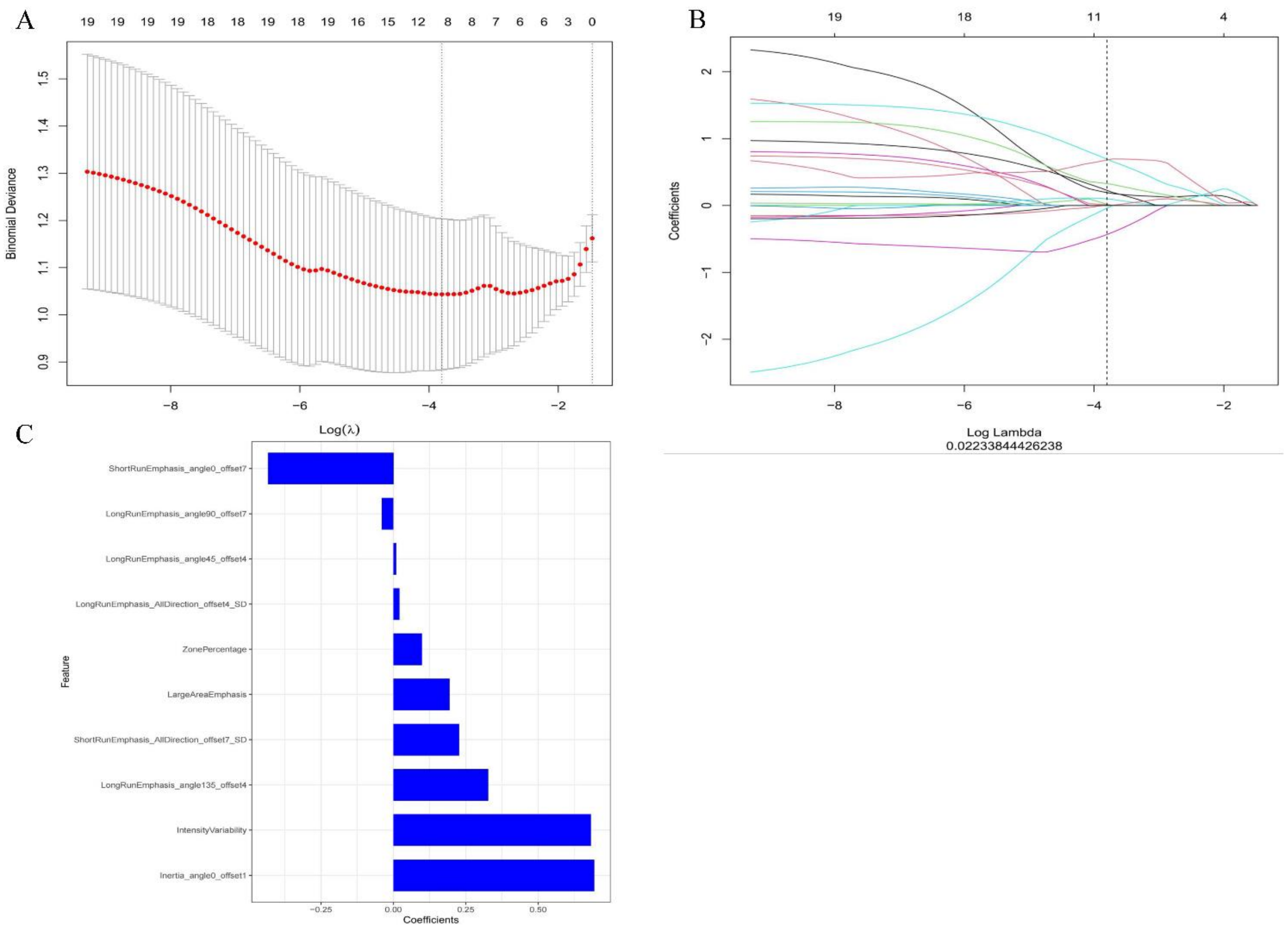

Supplementary Figure 2. Feature selection for first-RTLI radiomics model. (A) The error rate curve. (B) LASSO coefficient λ graph. Coefficient λ was selected in the LASSO using a 10-fold cross-validation. The coefficient λ with the lowest error rate was chosen. (C) The remaining features of T2WI after feature selection.

Note. RTLI = radiotherapy-induced temporal lobe injury, LASSO = least absolute shrinkage and selection operator, T2WI = T2 weighted imaging.

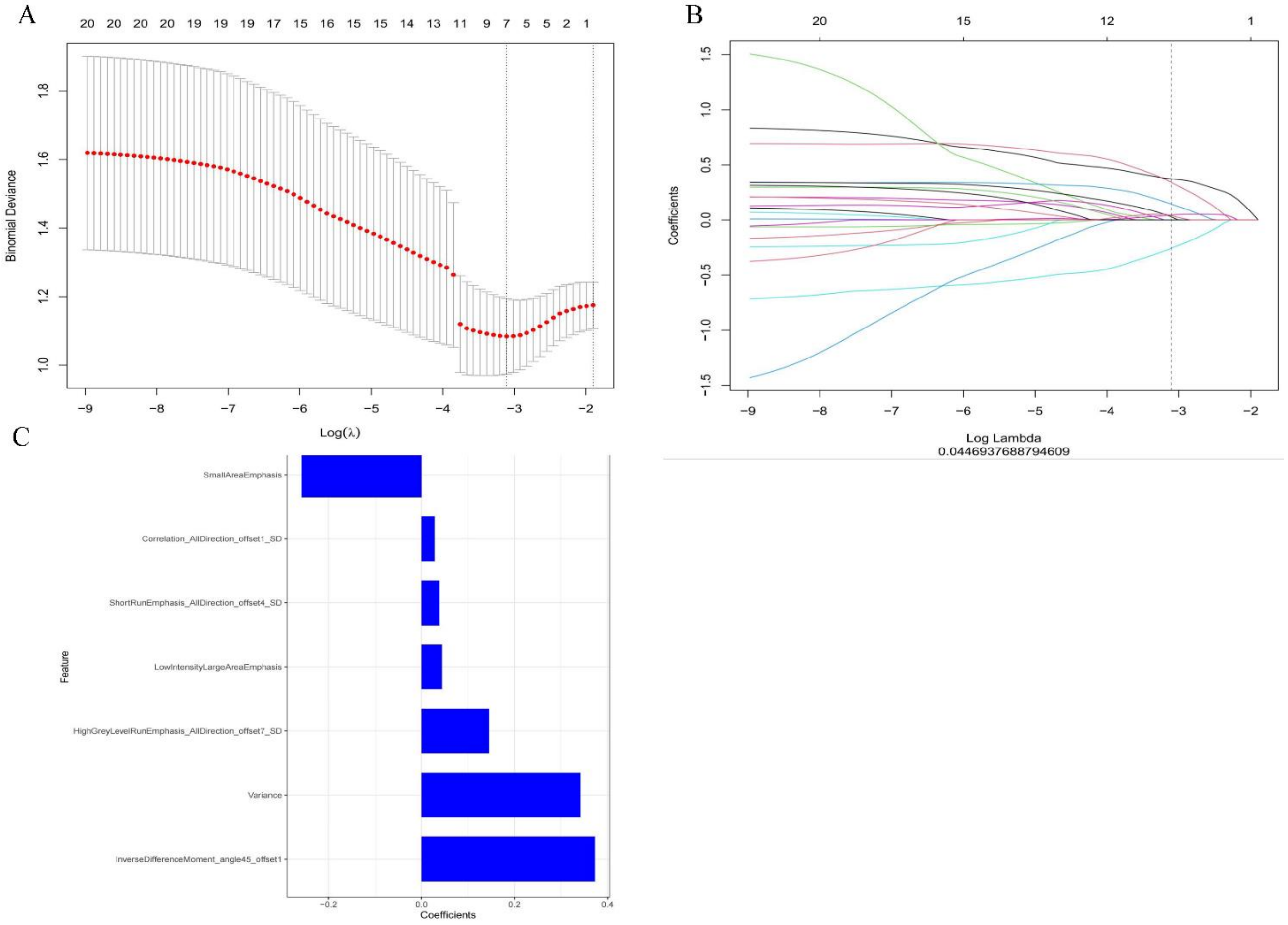

Supplementary Figure 3. Feature selection for delta radiomics model. (A) The error rate curve. (B) LASSO coefficient λ graph. Coefficient λ was selected in the LASSO using a 10-fold cross-validation. The coefficient λ with the lowest error rate was chosen. (C) The remaining features of T2WI after feature selection.

Note. LASSO = least absolute shrinkage and selection operator, T2WI = T2 weighted imaging.

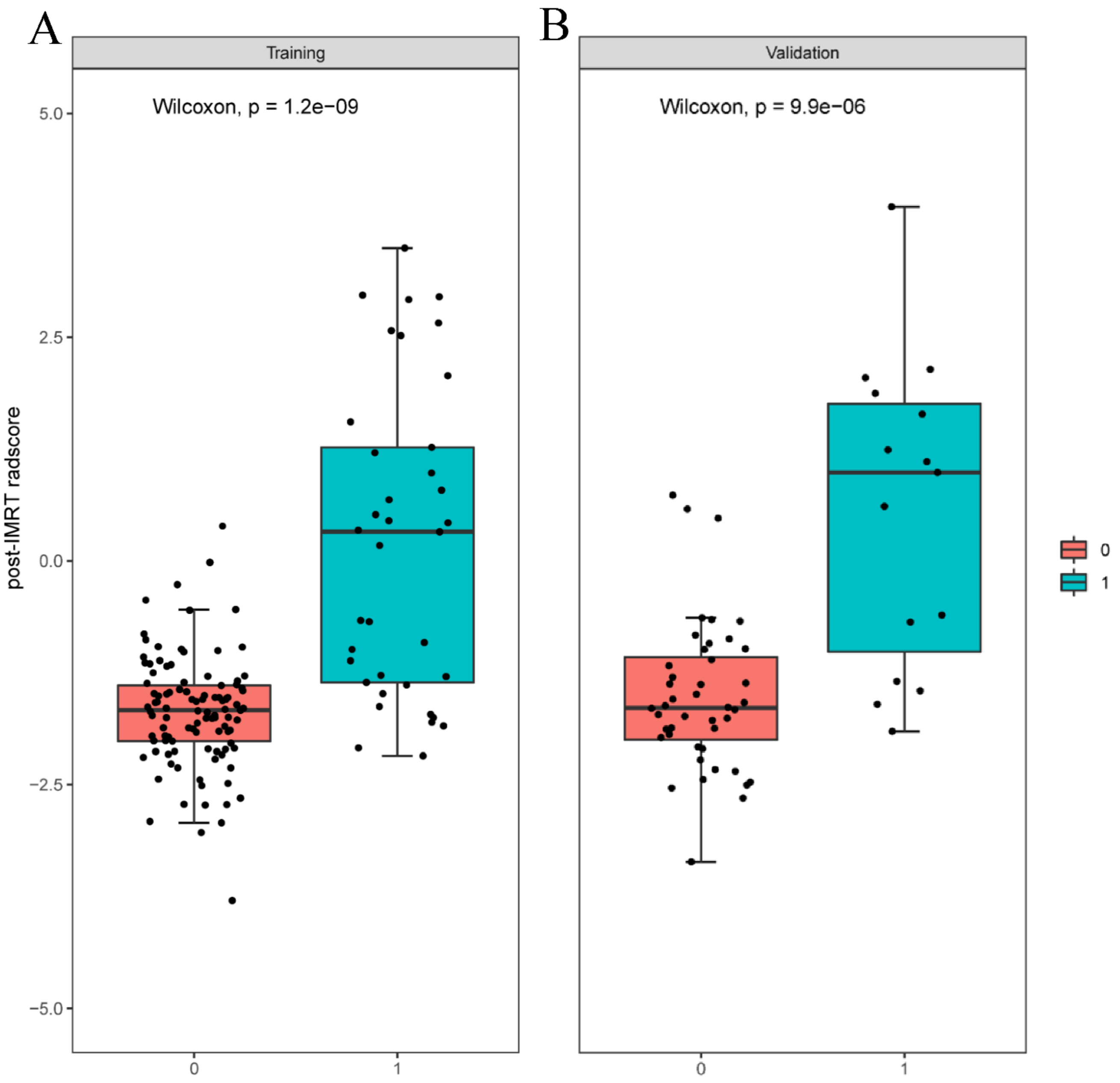

Supplementary Figure 4. Boxplots regarding post-IMRT radiomics radscore between the CBRN and non-CBRN groups in the training (A) and validation (B) sets.
Note. IMRT = intensity modulated radiotherapy, CBRN = cystic brain radionecrosis. (1: CBRN, 0: non-CBRN).

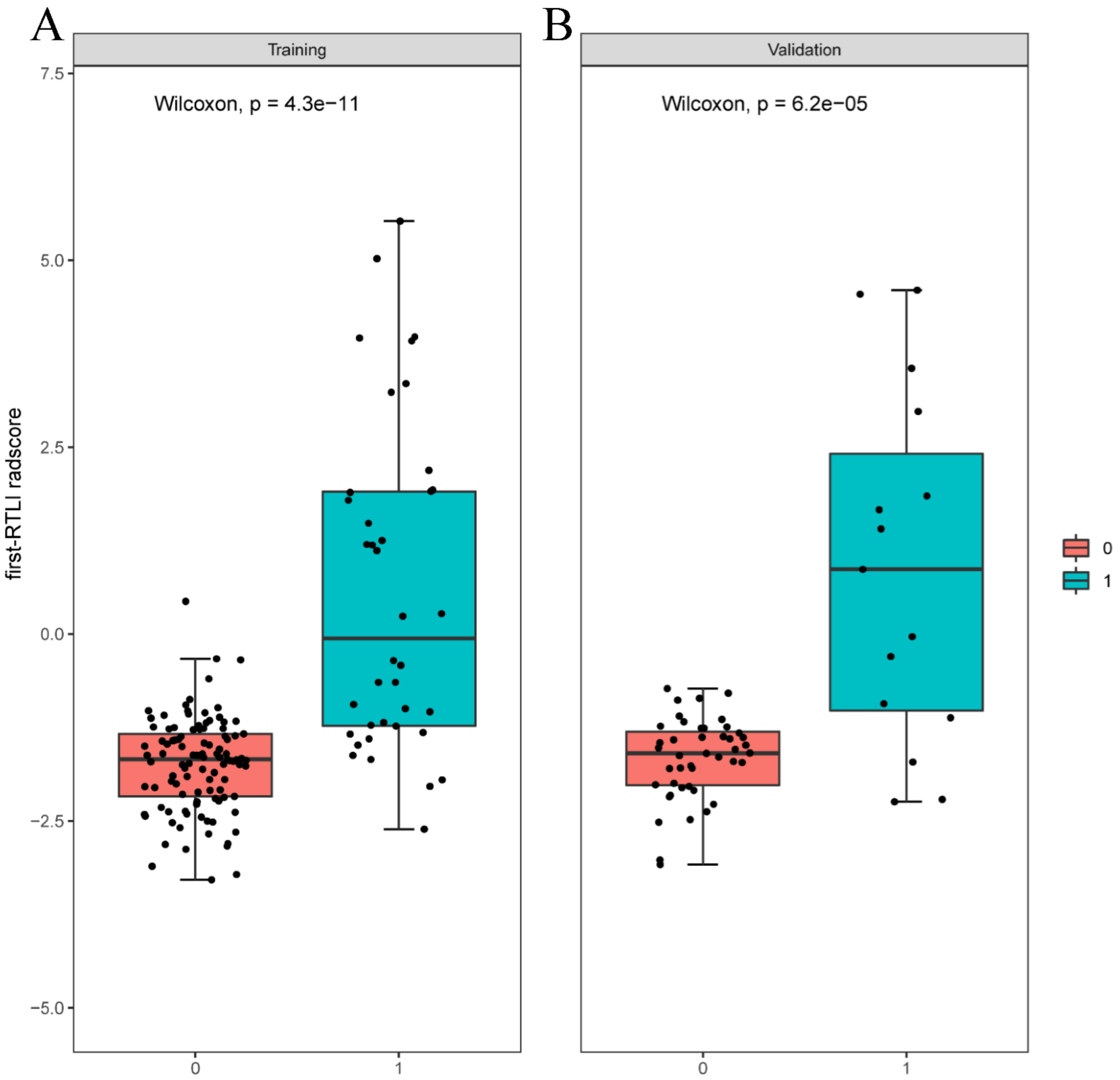

Supplementary Figure 5. Boxplots regarding first-RTLI radscore between the CBRN and non-CBRN groups in the training (a) and validation (b) sets.

Note. RTLI = radiotherapy-induced temporal lobe injury, CBRN = cystic brain radionecrosis. (1: CBRN, 0: non-CBRN)

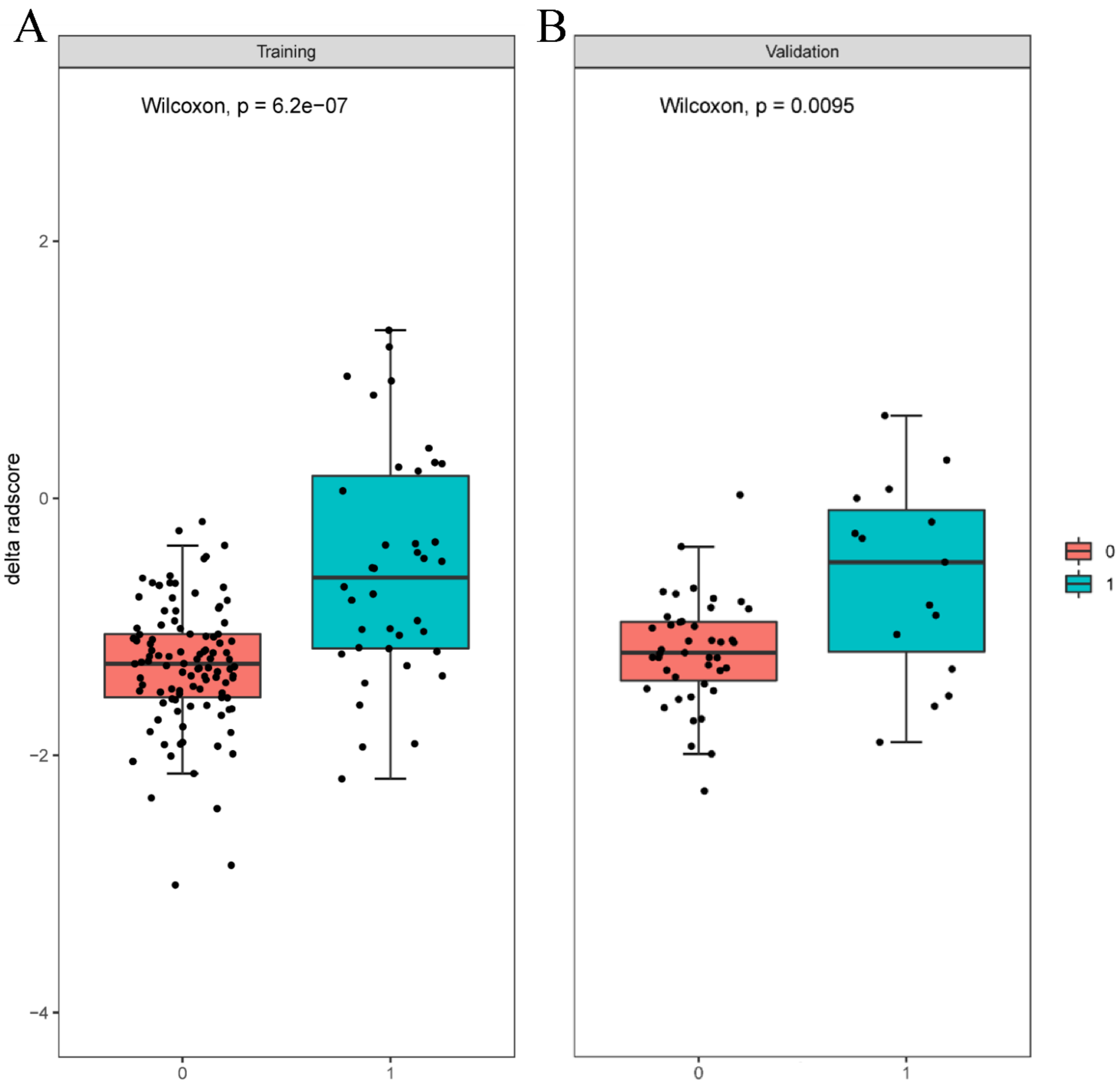

Supplementary Figure 6. Boxplots regarding delta radiomics radscore between the CBRN and non-CBRN groups in the training (a) and validation (b) sets.
Note: CBRN, cystic brain radionecrosis. (1: CBRN, 0: non-CBRN).

Supplementary Tables

Supplementary Table 1. The independent clinical risk factors of CBRN by multivariate logistic regression

Variables	β	Odds ratios	95% CI	*P*
Drinking	-1.178	0.17	(0.04, 0.75)	0.019

Note. CBRN = cystic brain radionecrosis

Supplementary Table 2. The post-IMRT radscore for CBRN and non-CBRN in the training and validation cohorts

Group	M (P_{25}, P_{75})	95% CI	Wilcoxon	
			Z	*P*
Training set				
CBRN	0.332(-1.368, 1.683)	-1.834(-2.409, -1.020)	-6.220	0.000
non-CBRN	-1.671(-2.027, -1.389)			
Validation set				
CBRN	0.989(-1.347, 1.871)	-2.232(-3.043, -0.990)	-4.143	0.000
non-CBRN	-1.644(-2.054, -1.109)			

Note. CBRN = cystic brain radionecrosis, IMRT = intensity modulated radiotherapy

Supplementary Table 3. The first-RTLI radscore for CBRN and non-CBRN in the training and validation cohorts

Group	M (P_{25}, P_{75})	95% CI	Wilcoxon	
			Z	*P*
Training set				
CBRN	-0.062(-1.256, 1.915)	-1.824 (-2.812, -1.101)	-6.595	0.000
non-CBRN	-1.677(-2.179, -1.312)			
Validation set				
CBRN	-0.862(-1.123, 2.974)	-2.455(-3.554, -1.074)	-3.812	0.000
non-CBRN	-1.595(-2.035, -1.279)			

Note. CBRN = cystic brain radionecrosis

Supplementary Table 4. The delta radscore for CBRN and non-CBRN in the training and validation cohorts

Group	M (P_{25}, P_{75})	95% CI	Wilcoxon	
			Z	*P*
Training set				
CBRN	-0.617(-1.552, -1.057)	-0.666(-0.937, -0.390)	-4.986	0.000
non-CBRN	-1.289(-1.175, 0.221)			
Validation set				
CBRN	-0.498(-1.475, -0.962)	-0.652(-1.029, -0.179)	-2.646	0.008
non-CBRN	-1.208(-1.331, -0.0004)			

Note. CBRN = cystic brain radionecrosis
